# Characteristics and Effectiveness of Physical Therapist-Supervised Exercise Interventions for Nursing Home Residents With Dementia: A Systematic Review

**DOI:** 10.1093/geroni/igae061

**Published:** 2024-06-20

**Authors:** Dennis Boer, Charlotte Schmidt, Shanty Sterke, Jan Schoones, Roy Elbers, Thea Vliet Vlieland

**Affiliations:** Physiotherapy, Kennemerhart, Haarlem, The Netherlands; Department of Orthopedics, Rehabilitation and Physical Therapy, Leiden University Medical Center, Leiden, The Netherlands; Research and Innovation, Kennemerhart, Haarlem, The Netherlands; Research Centre Innovations in Care, Rotterdam University of Applied Sciences, Rotterdam, The Netherlands; Physiotherapy, Aafje Nursing Homes, Rotterdam, The Netherlands; Directorate of Research, Leiden University Medical Center, Leiden, The Netherlands; Department of Physiotherapy, University of Applied Sciences Leiden, Leiden, The Netherlands; Department of Physiotherapy, University of Applied Sciences Leiden, Leiden, The Netherlands; Department of Orthopedics, Rehabilitation and Physical Therapy, Leiden University Medical Center, Leiden, The Netherlands

**Keywords:** Alzheimer’s disease, Long-term care, Physical therapy, Rehabilitation

## Abstract

**Background and Objectives:**

Although physical therapy, in particular exercise therapy, is widely used in nursing home residents with dementia, the literature on this topic is relatively scarce. This systematic review aimed to summarize the literature on the characteristics and effectiveness of exercise interventions supervised by physical therapists in nursing home residents with dementia.

**Research Design and Methods:**

Six electronic databases were systematically searched for relevant studies up to August 17, 2022. Randomized controlled trials (RCTs) comparing exercise interventions supervised by a physical therapist to any other form of intervention or usual care in nursing home residents with dementia were selected. Data were narratively analyzed and forest plots visualizing exercise effects were created.

**Results:**

From the 1 377 records retrieved and screened, 6 RCTs, reported in 11 papers, met the selection criteria. Included studies used multimodal or aerobic exercise interventions, with the frequency, duration and intensity varying across studies. Three of the 6 studies were at high risk of bias. Due to inconsistency in the findings and variety in outcome measures, results on the effectiveness of the interventions are inconclusive.

**Discussion and Implications:**

Our review emphasizes the need for more robust studies to offer understanding of the efficacy of exercise interventions supervised by physical therapists for nursing home residents with dementia.

**Translational Significance:** While exercise therapy, especially under the supervision of a physical therapist, is extensively employed for nursing home residents with dementia, its contents and effects have not been thoroughly investigated. We found 6 randomized controlled trials consisting of multimodal and aerobic exercise interventions targeting a variety of outcome measures. Because of inconsistencies in the results and the presence of bias, a conclusion regarding the effectiveness of the interventions could not be reached. By identifying gaps and emphasizing the need for methodologically robust studies, this review contributes to the development of interventions that can positively affect the well-being of nursing home residents with dementia.

## Background and Objectives

Dementia has an estimated prevalence of 48%–84% (1,2) in nursing home residents in Western countries. Nursing home residents often display multiple geriatric syndromes that result in limited functional performance and mobility and increased care needs (3). Physical exercise is an important intervention to positively affect their functional capacities, thereby improving daily functional performance (4).

Exercise guidelines (4–6) have been published for nursing home residents, including those with dementia. Recommendations advocate the use of multicomponent exercise consisting of strength, endurance, and balance exercises to be conducted a minimum of 2 times per week at moderate intensity, under the supervision of an exercise specialist (4). In this context, physical therapy services are globally used by 10%–67% of nursing home residents (7). The usage, however, is unevenly spread, with residents without dementia and on temporary stay receiving significantly more physical therapy (8,9). A rationale for this is unclear, and may result in permanent residents with dementia not receiving sufficient physical therapy (8).

Of the 6 known systematic reviews (10–15) on exercise interventions in nursing home residents with dementia, 2 (10,14) have included studies with physical therapist-supervised exercise interventions, while in the other reviews (11–13,15) supervisors were not described. Apart from physical therapists, the interventions were conducted by research assistants (10,14), occupational therapists (10,14), psychology students (10,14), recreational therapists (10), nurses (10,14), caregivers (10,14), and an exercise scientist (14). Furthermore, the characteristics of the included interventions varied widely. Reviews included both traditional physical exercise interventions as well as dance (10), hand movement (10), walking and talking (10,12,14,15), Tai Chi (12), and ball games (14). This variation in both exercise supervisors and intervention characteristics limits the translation of findings into clinical care.

Regarding the effects of exercise, earlier reviews evaluated the impact of interventions on a wide range of outcomes. While 2 reviews, specifically targeting depression (12) and cognition (13), identified potential positive effects, the overall evidence presents conflicting or limited findings on the outcomes independence of daily activities (activities of daily living [ADL]) (10,15), walking performance and endurance (10,14,15), cognition (10), depression (10,15), behavioral symptoms (10,15), nutrition (15), mobility (10,14), and balance (14). Explanations for this uncertainty of the effects might stem from methodological flaws of included studies (14,15), as well as the aforementioned variability in intervention characteristics across studies (10,13).

A systematic review concentrating exclusively on exercise interventions supervised by physical therapists could reduce the heterogeneity in exercise characteristics. This approach is more in line with the clinical setting of a nursing home, where physical therapists often oversee exercise programs. In terms of effectiveness, a prior study has shown that the involvement of an exercise specialist improves exercise adherence and intensity, which may potentially lead to better health outcomes for this population (16).

To summarize, exercise under the supervision of an exercise specialist, such as a physical therapist, is recommended by international guidelines (4,5). Although physical therapy is frequently used in the treatment of nursing home residents with dementia, its effects are uncertain. Therefore, the aim of the present systematic review was to systematically search the available literature and answer the following questions:

What are the characteristics of exercise interventions supervised by physical therapists for nursing home residents with dementia as employed in (cluster) randomized controlled clinical trials?
What is the effectiveness of exercise interventions compared to usual care or any other intervention?

## Research Design and Methods

### Study Design

This systematic review was registered in the PROSPERO prospective register of systematic reviews (registration number: CRD42022351596, link: https://www.crd.york.ac.uk/prospero/display_record.php?RecordID=351596) and is reported according to Preferred Reporting Items for Systematic Reviews and Meta-Analyses guidelines (17).

### Search Strategy

The search strategy was designed collaboratively by 3 authors (D.B., T.V.V., and J.S.), one of whom is a trained librarian (J.S.). The search strategy was developed for PubMed/Medline and was then modified for Cochrane Library, Embase, Web of Science, Emcare, and the Physiotherapy Evidence Database (PEDro), using MeSH terms and free text. Databases were searched from inception up to the current date (August 17, 2022). Key PI(M)CO terms included “nursing homes,” “dementia,” “exercise therapy,” and “randomized controlled trials.” The full search strategy can be found in Supplementary Table 1. The database of clinicaltrials.gov was searched for ongoing studies or unpublished data. Previously published systematic reviews and the reference lists of the included articles were manually searched to identify eligible articles.

### Selection of Studies

Retrieved records were exported to the Rayyan review software (Rayyan Systems Inc., Cambridge, MA). After elimination of duplicates, selection of studies was independently performed by 2 reviewers (D.B., C.S.). Studies were eligible for inclusion if they included nursing home residents with a diagnosis of dementia irrespective of the specific diagnostic criteria. Only (cluster) randomized controlled trials (RCTs) were considered in order to reduce the heterogeneity among studies. Studies were included if they compared an exercise intervention (fully or partially supervised by a physical therapist) to any other intervention or no intervention. Articles written in English, Dutch, or Spanish were considered. Studies were excluded if they included patients with dementia who temporarily stayed in a nursing home for rehabilitation; included mixed populations of residents with and without dementia and did not report separately on residents with dementia; concerned an intervention where physical therapy was part of a multicomponent intervention (eg, a fall-risk program with medication provision, exercise therapy, and home adaptations). Selection of studies was conducted in 2 steps. First, titles and abstracts were screened using the abovementioned criteria, and if deemed relevant or when eligibility was unclear, full-text papers were obtained. Full-text papers were subsequently assessed for eligibility using the same criteria. A third independent reviewer (T.V.V.) was consulted in case of discrepancies. Study protocols were reviewed to determine if separate articles belonged to the same study.

### Data Extraction

Two reviewers (D.B., S.S.) extracted all data from eligible studies independently according to a prespecified data extraction sheet in Microsoft Excel (Version 2202 14931.20626). In a meeting, the 2 researchers discussed their individual extracted data to reach consensus.

The following study characteristics were extracted: study type, age, sex, type of dementia, the nature of the treatment arms, and duration of study/follow-up. Extraction of intervention characteristics was based on 2 templates for the description of nonpharmacological/exercise interventions, that is, the CERT template (18) and the TIDieR checklist (19). The characteristics considered in this review consisted of exercise type; materials used; procedures; exercise conductors; group size; place of delivery; intervention frequency and duration; tailoring; methods of assessing adherence, adverse events; and study length. Regarding the extraction of outcomes, no primary outcomes of interest were defined for this review. We extracted all outcome data (any measures of effectiveness and/or safety) as presented in the studies, including within-group and between-group difference, confidence interval, (interquartile) range, standard deviation (*SD*), and/or *p* value, where appropriate.

### Risk of Bias Assessment and Assessment of Certainty in the Evidence

The risk of bias of individual studies was assessed with the most recent version of the Cochrane risk-of-bias tool for randomized trials (RoB 2) (20), or the adapted risk-of-bias tool for cluster-randomized trials (21). The RoB 2 tool assesses bias across 5 domains: randomization; deviations from the intended intervention; missing outcome data; measurement of the outcome; selection of reported results (publication bias). The risk of bias outcome is labeled as “low,” “some concerns,” or “high.” In exercise interventions, it is nearly impossible to blind participants and people who deliver the intervention. Therefore, these criteria were not considered. Two reviewers (D.B., C.S.) independently assessed the risk of bias of included studies. A third assessor (T.V.V.) was available if discrepancies could not be resolved.

The Grading of Recommendations, Assessment, Development, and Evaluation (GRADE) approach (22) was used to assess the certainty in the evidence of exercise effects on the outcomes reported. The certainty in the evidence was determined for outcomes with a minimum of 3 studies reporting on it. Certainty was categorized into “High” (high confidence in the found effect), “Moderate” (future research could have an important impact in the estimated effect), “Low” (future research is very likely to have an important effect), and “Very low” (any estimate of effect is very uncertain). According to the GRADE approach, certainty is initially determined by study design (RCTs have a higher initial quality compared to observational studies) and may be affected by factors such as risk of bias, inconsistency, indirectness, imprecision, and publication bias. It can be positively influenced by a large effect, dose–response, or confounding that reduces the observed effect.

### Data Analysis

The Synthesis Without Meta-analysis guideline (23) and the Cochrane Handbook for Systematic Reviews of Interventions (24) were used for the narrative synthesis of the data. Due to the high degree of heterogeneity in outcomes and measurements in the studies, a meta-analytic approach was not appropriate. To provide an overview of the results from the individual studies, forest plots with standardized effect sizes were created. The R environment for statistical computing (version 4.2.2) and the package *Metafor* (25) were used to create the forest plots. The mean postinterventions scores of the intervention and control groups and their *SD*s were used to calculate a standardized mean difference (Hedges *G*) for each study. In cases where negative mean scores indicated a positive effect, scores were multiplied by −1 to adjust direction of effect in forest plot. If mean postintervention scores were not available for a study, the change score and corresponding *SD* were used. If necessary, median and interquartile ranges were converted to means and confidence intervals according to the suggested method in the Cochrane handbook (26).

## Results

### Selection of Studies

**Figure 1.**
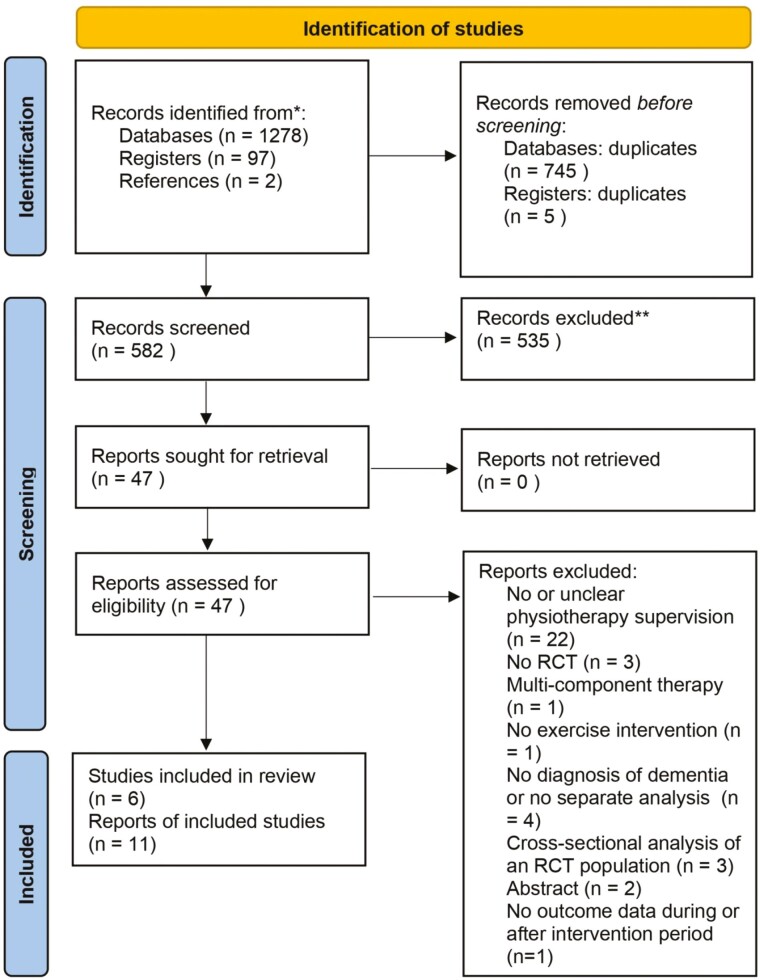
Flowchart of the selection process of the studies. RCT = randomized controlled trial.

We identified 1 278 records from databases, 97 from the clinicaltrials.gov register, and 2 after screening the included studies and reference lists of previously published systematic reviews. After removing 750 duplicates, 581 titles and abstracts were screened, resulting in the retrieval of 46 full-text articles. From the 46 full-text articles screened, 11 articles reporting findings from 6 studies were eventually included in this systematic review (Figure 1). Authors of 5 studies were contacted. One author (27) provided additional information on the type of dementia of the participants; 2 authors provided (28,29) information on the data analysis and study outcomes. The other 2 authors (10,30) did not reply or were not able to act to our request for additional information on effect estimates. See Supplementary Table 2 for the list of full-text screened but excluded articles.

### Study Characteristics

The main characteristics of the studies are presented in Table 1. The outcomes of 1 study were presented in 4 separate articles (29,31–33). In 2 studies, the outcomes were presented in 2 separate articles each (34–37), and 3 studies presented all outcomes in 1 article (27,28,30). To ensure clarity in this review, we will cite the first published article when discussing study characteristics. There were 2 cluster-randomized controlled trials (28,29,36), and 4 traditional RCTs (27,30,34). The intervention length ranged from 12 weeks (34) to 15 months (30), the number of participants from 24 (27) to 191 (36), and mean average age from 83 (27) to 87 years (28). One study (34) included 2 intervention groups that are both included in this review. In 3 studies (28,29,36), the control group performed light sitting recreational activities; in the other 3 studies (27,30,34), the control group received usual care.

**Table 1.** Main Study Characteristics of the 6 Included Studies

Study	Study design	Duration of study	Number of participants	Age, mean (*SD*)	Female, *n* (%)	Baseline cognitive function, mean (*SD*)	Baseline physical independence, mean (*SD*)	Number and nature of treatment arms
Toots et al. (29,31,32), Bostrom et al. (33)	Cluster RCT	4 months	IG: 92 CG: 92	Total: 85.1 (7.1) IG: 84.4 (6.2) CG: 85.9 (7.8)	Total: 141 (75.8%) IG: 70 (75.3%) CG: 71 (76.3%)	MMSE (0–30) Total: 14.9 (3.5) IG: 15.4 (3.4) CG: 14.4 (3.5)	Barthel Index (0–20) Total: 10.9 (4.4) IG: 10.7 (4.5) CG: 11.0 (4.4)	IG: high-intensity functional weight-bearing exercise program CG: sitting activities (conversing, singing, picture viewing, listening to readings or music)
Brett et al. (34,35), Australia	RCT	12 weeks	IG 1: 17 IG 2: 19 CG: 19	Total: 85 (range 58–100) IG 1: 86 IG 2: 84 CG: 86	Total: 36 (66%) IG 1: 13 (76%) IG 2: 13 (68%) CG: 10 (53%)	—	—	IG 1: multimodal exercise intervention IG 2: short-duration, high-frequency multimodal exercise intervention CG: usual care
Cancela et al. (30), Spain	RCT	15 months	IG: 73 CG: 116	IG: 80.63 (8.32) CG: 82.90 (7.42)	IG: 81% CG: 44%	MMSE (0–30) IG: 14.9 (2.4) CG: 15.2 (2.5)	Katz Index (0–6) IG: 4.3 (0.9) CG: 4.3 (1.0)	IG: very low resistance aerobic cycling program CG: usual recreational activities
Littbrand et al. (36), Conradsson et al. (37), Sweden	Cluster RCT	13 weeks	IG: 91 CG: 100	Total: 84.7 (6.5) IG: 85.3 (6.1) CG: 84.2 (6.8)	Total: 139 (73%) IG: 67 (74%) CG: 72 (72%)	MMSE (0–30) Total: 17.8 (5.1) IG: 17.5 (5.0) CG: 18.0 (5.3)	Barthel Index (0–20) Total: 13.1 (4.2) IG: 12.8 (4.5) CG: 13.4 (3.8)	IG: high-intensity functional weight-bearing exercise program Postintervention: daily functional tasks CG: sitting activities (watching films, singing, reading, and conversation)
Telenius et al. (28), Norway	Cluster RCT	12 weeks	IG: 87 CG: 83	Total: 86.7 (7.4) IG: 86.9 (7.0) CG: 86.4 (7.8)	Total: 74% IG: 59 (68%) CG: 61 (73%)	MMSE (0–30) Total: 15.7 (5.0) IG: 15.6 (5.0) CG: 15.8 (5.0)	Barthel Index (0–20) Total: 13.5 (3.6) IG: 13.6 (3.5) CG: 13.4 (3.6)	IG: high-intensity functional weight-bearing exercise program CG: light physical activity, reading, playing games, listening to music and conversations
Venturelli et al. (27), Italy	RCT	24 weeks	IG: 12 CG: 12	IG: 83 (6) CG: 85 (5)	86% (total sample, before exclusion and randomization)	MMSE (0–30) IG: 13 (2) CG:12 (2)	Barthel Index (0–100) IG: 34 (4) CG:35 (6)	IG: supervised walking at fastest possible pace CG: usual care

*Notes*: CG = control group; IG = intervention group; MMSE = Mini-Mental State Examination; RCT = randomized controlled trial; *SD* = standard deviation.

### Characteristics of Interventions

Table 2 presents detailed information on the characteristics of the exercise interventions.

**Table 2.** Intervention Characteristics of the 6 Included Studies Based on the CERT and TIDieR Templates

Study	Exercise type	Materials	Procedures and processes	Supervisors	Individual or group	Place of delivery	Frequency and duration	Tailoring	Method of measuring adherence	Method of measuring intensity	Method of measuring adverse events
Toots et al. (29,31,32), Bostrom et al. (33)	Strength, balance	Weighted (safety) belts, steps, chairs, cushions, mattresses, balls, bean bags	HIFE^a^	Two PTs	Group; 3–8 participants. Individually supervised sessions for participants unable to attend the group sessions	16 residential care facilities in Sweden	Five sessions of 45 min per 2 weeks	Yes	Evaluated after each session on a predefined scale (28)	Evaluated after each session on a predefined scale (28)	Active monitoring after each session: (1) minor and temporary; (2) serious symptoms; (3) manifest injury or disease; (4) death (28)
Brett et al. (34,35)	Strength, balance, endurance, flexibility	Hand weights, balls, cones, static pedals	Warming up, strength, balance, aerobic exercises, cooling down	One PT	Group; maximum of 5 participants	Two NHs in Australia; intervention was held in a noise-adjustable sitting room	Frequency: intervention 1: one 45-min session/week; intervention 2: three 15-min sessions/week	Yes	Observation of slight breathlessness	Not reported	Not reported
Cancela et al. (30)	Aerobic	Recumbent bicycle geared to low resistance	Minimum of 15-min exercise on self-selected pace on very low resistance	PT	Individual or in pairs	Elderly home care facilities in Galicia (Spain); intervention took place in the gymnasium	Daily for a minimum of 15 min	Yes	PT monitored each session and registered the time each individual exercised	Not reported	Not reported
Littbrand et al. (36), Conradsson et al. (37)	Strength, balance after group intervention: physical tasks integrated into daily life	Weighted (safety) belts, steps, chairs, cushions, mattresses, balls, bean bags	HIFE	Two PTs	Group; 3–9 participants	Nine residential care facilities in Sweden	Frequency: 5 sessions of 45 min per 2 weeks	Yes	Not reported	Evaluated after each session on a predefined scale (28)	Not reported
Telenius et al. (28)	Strength, balance	Weighted (safety) belts, steps, chairs, cushions, mattresses, balls, bean bags	HIFE	One PT per 3 participants	Group; 3–6 participants	18 NHs in Norway	Frequency: 2 sessions of 50–60 min per week	Yes	Not reported	PTs documented the intensity after each session	Not reported
Venturelli et al. (27)	Aerobic	At the end of the session, cookies were offered to the NH resident and caregiver	Some minutes of informal chatting before the start of the exercise	Caregivers (staff and family)	Individually, guided by a caregiver	Alzheimer care unit in Italy; walking sessions were conducted in the hallway.	Frequency: minimum of 30 min, 4 times/week	Yes	Walking times and distance were recorded and checked before and after each visit.	Walking on the participant’s own but fastest pace	Not reported

*Notes*: NH = nursing home; PT = physical therapist.

^a^The HIFE (High-Intensity Functional Exercises) program involves: a 5-minute warm-up for upper and lower extremities while sitting, at least 2 lower-limb strength exercises and 2 balance exercises in 2 sets.

#### Type, materials used, and procedures

In 4 studies (28,29,34,36), multimodal exercise interventions (interventions comprised of more than 1 exercise type) and in 2 studies (27,30), aerobic exercise interventions were used. Regarding multimodal exercise interventions, 3 studies (28,29,36) employed the same HIFE (High-Intensity Functional Exercises) program (38). The HIFE program is a high-intensity multimodal group exercise intervention that, after a warming up, focuses on lower-limb strength and balance exercises. The program uses weighted (safety) belts, steps, chairs, cushions, mattresses, balls, and bean bags. In another study (34), the intervention comprised strength, balance, endurance, and flexibility exercises and used static bike trainers, hand weights, balls, and cones as materials. Participants performed a warming up and cooling down before and after each exercise session. In the study by Venturelli et al. (27), the intervention comprised an aerobic type of exercise intervention in the form of supervised walking. In their study procedure, cookies were offered to the resident and caregiver after the exercise session as a positive psychological reinforcement. In the study by Cancela et al. (30), recumbent stationary bicycles were used, where participants performed aerobic exercise in the form of cycling on a very low resistance.

#### Exercise supervisors and group size

Four studies (28,29,34,36) evaluated small-group exercise interventions supervised by 1 or 2 physical therapists. Individual sessions for participants unable to attend the group sessions were offered in one (29) of those studies. In another study (30) participants performed exercises individually or in pairs, supervised by a physical therapist. In the study by Venturelli et al. (27), the intervention comprised individual walking sessions provided by caregivers (nursing staff and family caregivers) with the physical therapist giving instructions regarding walking speed, intensity, and distance. Family caregivers were not involved in any other studies.

#### Place of delivery and tailoring

All interventions took place in long-term care facilities, described as nursing homes (28,34), residential care facilities (29,36), elderly home care facility (30), or Alzheimer care unit (27). In 2 studies, it was specified where the intervention took place (noise-adjustable sitting room (34) and gymnasium (30)). All 6 studies reported tailoring the exercises to the participants’ functional capacities.

#### Intervention frequency and duration

The study by Brett et al. (34) included 2 intervention groups: one group exercised once per week for 45 minutes, while the other group exercised 3 times per week for 15 minutes per session. In 2 studies (29,36), the exercise group exercised 5 times per 2 weeks, with sessions lasting 45 minutes. In another study (28), the exercise group exercised 2 times per week for 50–60 minutes per session. In the study by Cancela et al. (30), participants exercised daily for a minimum of 15 minutes, and in the study by Venturelli et al. (27), the participants exercised at least 4 times per week, with sessions lasting a minimum of 30 minutes.

#### Methods of measuring adherence, intensity, and adverse outcomes

The measurement of adherence was reported in 2 studies (27,29). One study (29) referred to a predefined scale (38), while in the other study, adherence was monitored by recorded walking times and distance (27). Regarding exercise intensity, in 3 studies (28,29,36), the exercise intensity was evaluated on a scale that distinguished high, moderate, and low. Another study (34) described that intensity was monitored by observing if participants experienced slight breathlessness. One (29) of the 6 studies described the methodology for defining and monitoring adverse events. That study referred to a protocol (38) in which adverse events were actively monitored during and after each session, whereas the severity of any occurring event was categorized into: minor and temporary, serious symptoms (potential risk of severe injury or life-threatening), manifest injury or disease, or death.

#### Results of exercise adherence, intensity, and adverse outcomes

Adherence was reported in all studies, and ranged from 72% (36) to 93.4% (27) in the intervention group, and from 69% (28) to 70% (34) in the social activities control groups. Regarding the intensity of exercise, 3 studies (28,29,36) included exercises at a high-intensity level, 1 study (34) employed exercises at moderate intensity, 1 (30) at very light intensity, and 1 (27) at the participant’s own, but fastest, pace. Regarding adverse events, 1 study (29) reported that all adverse events were minor and temporary, 2 studies (27,30) reported that no adverse events were related to the exercise program, the other 3 studies reported that there were no adverse events (28,34) or that no adverse event resulting in injury, disease, or death (36).

### Outcomes of Multimodal Exercise Interventions

**Figure 2.**
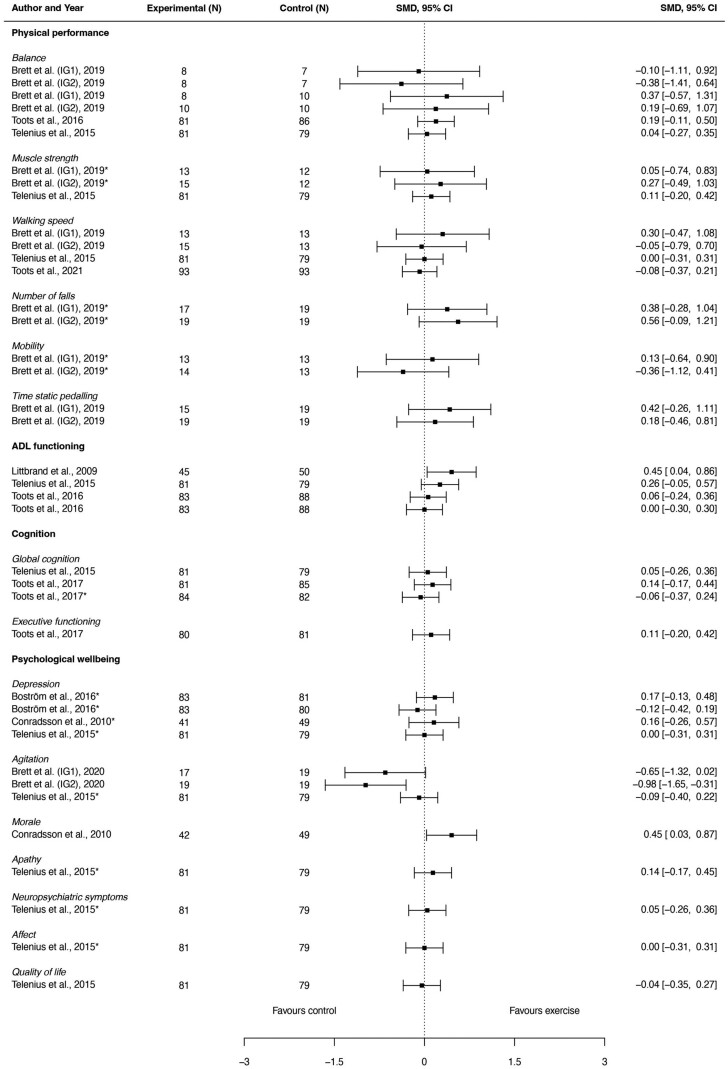
Visual summary in the form of a forest plot of the effect sizes and confidence intervals of individual studies that employed multimodal exercise interventions for all study outcomes. ADL = activities of daily living.

Exercise effects of multimodal exercise interventions were evaluated on a total of 25 different outcomes, which we categorized in “physical performance,” “ADL functioning,” “cognition,” and “psychological well-being.” Figure 2, in the form of a forest plot, provides a visual summary of the effect sizes and confidence intervals of individual studies that employed multimodal exercise interventions for all study outcomes. Further details on the outcomes can be found in Supplementary Table 3.

#### Physical performance

Three studies (28,29,34) evaluated the effects on physical performance outcome measures. Outcome measures and the number of studies that reported on physical performance were: balance (3) (28,29,34), falls (1) (34), mobility (1) (34), muscle strength (2) (28,34), timed static pedaling (TSP; 1) (34), and walking speed (3) (28,32,34). Two studies (28,29) found a significant positive effect of multimodal exercise on balance, when compared to sitting recreational activities; 1 study (34) found a significant positive effect on falls, when compared to usual care. No significant effects were found on mobility, muscle strength, TSP, and walking speed.

#### ADL functioning

Three studies evaluated the effects on ADL functioning (28,29,36). All studies used the Barthel Index (BI); 1 study (29) additionally used the Functional Independence Measure. One study (36) reported a positive exercise effect compared to sitting recreational activities, while 2 studies (28,29) found no effect.

#### Cognition

Two studies (28,31) evaluated the effects on cognition. Outcome measures and the number of studies reporting on it were global cognition (2) (28,31) and executive functioning (1) (31). Both studies compared multimodal exercise to sitting recreational activities, and found no significant effects.

#### Psychological well-being

Four studies (28,33,35,37) evaluated the effects on psychological well-being. Outcome measures and the number of studies were depression (3) (28,33,37), agitation (2) (28,35), morale (1) (37), apathy (1) (28), behavioral and neuropsychiatric symptoms (1) (28), affect (1) (28), and quality of life (1) (28). Positive effects were found on the outcomes morale (37) and apathy (28) compared to sitting recreational activities. No effect was found on other outcomes.

### Outcomes of Aerobic Exercise Interventions

**Figure 3.**
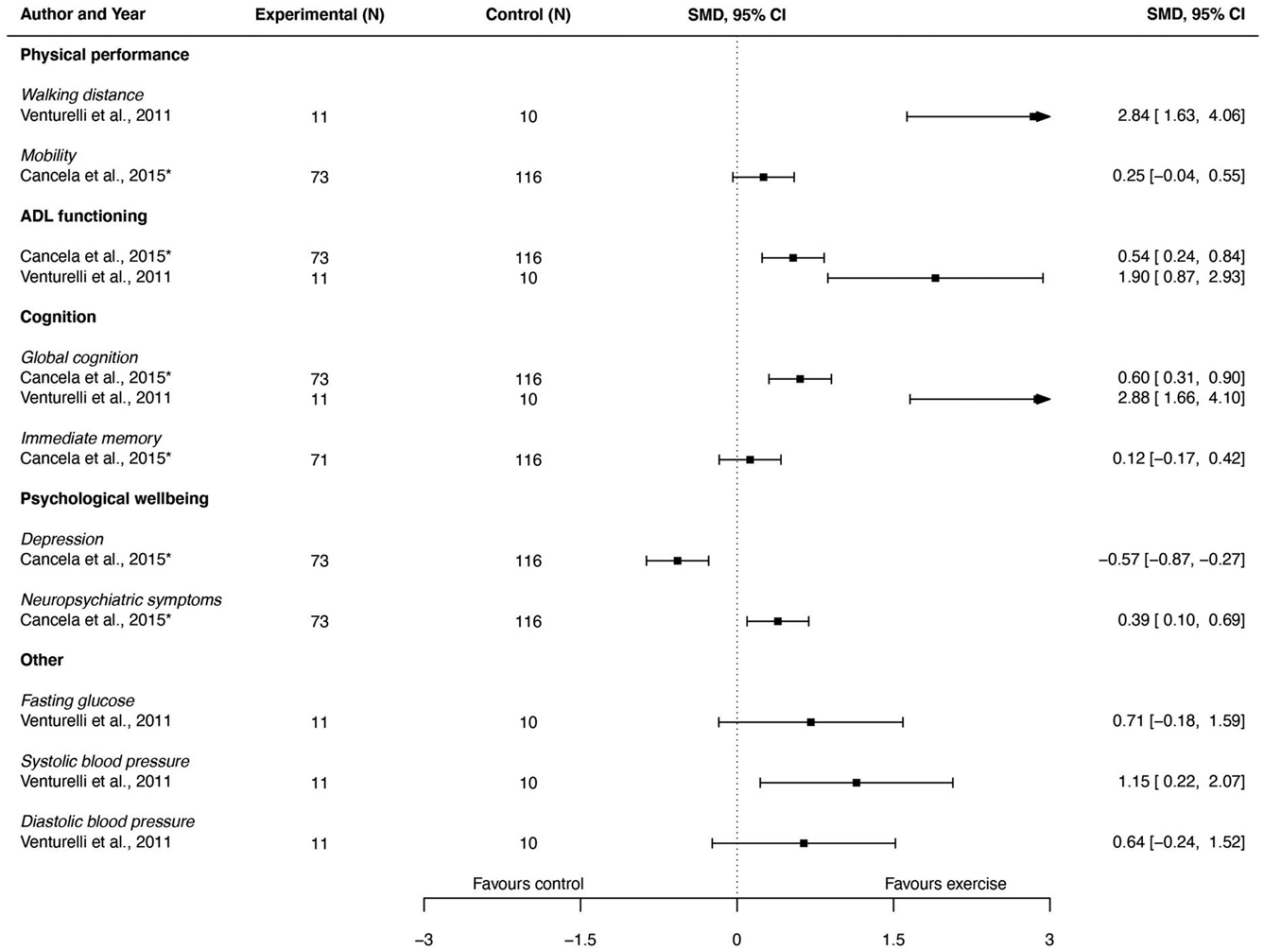
Visual summary in the form of a forest plot of the effect sizes and confidence intervals of individual studies that employed aerobic exercise interventions for all study outcomes. ADL = activities of daily living.

Aerobic exercise effects were evaluated on a total of 11 different outcomes, which were categorized in “physical performance,” “ADL functioning,” “cognition,” “psychological well-being,” and “others.” Figure 3, in the form of a forest plot, provides a visual summary of the effect sizes and confidence intervals of individual studies that employed aerobic exercise interventions for all study outcomes. Further details on the outcomes can be found in Supplementary Table 3.

#### Physical performance

Two studies (27,30) evaluated the effects on physical performance. One study (30) evaluated the effect of aerobic exercise on mobility, the other on walking distance (27). In both studies, significant positive effects were found.

#### ADL functioning

Two studies evaluated the effects on ADL functioning with 1 study using the Katz Index (30) and 1 study using the BI (27). In both studies, significant positive effects were found.

#### Cognition

Two studies (27,30) evaluated the effects on cognition. One study (30) measured global cognition and immediate memory; 1 study (27) measured global cognition only. Positive effects were found on global cognition (27,30) and on immediate memory (30).

#### Psychological well-being

One study (30) evaluated the effects on psychological well-being. It evaluated the effects on depression and neuropsychiatric symptoms. No significant effects were found.

#### Others

One study (27) evaluated the effects on the outcomes glycemia and blood pressure (systolic and diastolic). No significant effects were found.

### Risk of Bias

Assessment of the risk of bias in the 6 included studies is presented in Table 3. Three of the 6 studies (27,30,34) were considered to be at high risk of bias. Causes of a high risk of bias were regarding an inappropriate analysis (27,34), missing outcome data (30,34), no assessor blinding (27,30,34), and the selection of the reported result (27,30). One study (28) had some concerns regarding risk of bias due to missing outcome data. Two studies (29,36) were considered at a low risk of bias. Additionally of the risk of bias screening, sources of funding for the included studies were investigated. No conflicting interests were found.

**Table 3.** Assessment of the Risk of Bias in the 6 Included Studies

Study	Randomization process	Deviations from intended interventions	Missing outcome data	Measurement of the outcome	Selection of the reported result	Overall
Toots et al. (29,31,32); Boström et al. (33)	1	1	1	1	1	1
Brett et al. (34,35)	1	2	2	2	1	3
Cancela et al. (30)	1	1	2	2	2	3
Littbrand et al. (36); Conradsson et al. (37)	1	1	1	1	1	1
Telenius et al. (28)	1	1	2	1	1	2
Venturelli et al. (27)	1	2	1	3	3	3

*Notes*: 1 = low risk of bias; 2 = some concerns; 3 = high risk of bias.

### Certainty in the Evidence

Certainty in the evidence was determined separately for multimodal exercise interventions regarding outcomes with a minimum of 3 studies reporting on it. The outcomes physical performance (balance (28,29,34) and walking speed (28,32,34)), ADL functioning (28,29,36), and depression (28,33,37), all had 3 studies reporting on its respective outcome. For all outcomes, the certainty in the evidence was determined as very low. Causes for low certainty in the evidence could be attributed to risk of bias (balance, walking speed, ADL functioning, depression), inconsistency in the results (balance, ADL functioning, depression), indirectness in the results (balance, walking speed), and imprecision in the results (balance, walking speed, depression). Supplementary Table 4 provides more detail on the determination of the certainty in the evidence.

## Discussion

### Summary

Previous systematic reviews (10–15) on exercise interventions for nursing home residents with dementia included all kinds of exercise interventions, regardless of their supervision. Although some previous reviews (10,14) included physical therapist-supervised exercise interventions, none synthesized their characteristics and effectiveness.

Regarding study characteristics, the studies included in our review showed variation in population size, length, and composition of the intervention and the outcome measures used. Regarding intervention composition, exercise with strength, balance, and aerobic modalities at moderate or high intensity for at least 30–45 minutes, 2–3 times per week is recommended by dementia organizations and international geriatric working groups (4–6). None of the interventions in the studies in the present systematic review fulfilled these recommendations. When it comes to study length, in 3 (28,34,36) of the 6 studies, the length of the intervention was not longer than 13 weeks. To our knowledge, no minimal duration of exercise length has been determined. However, a review on physical activity, cognition, and brain plasticity (39) has suggested an exercise length of 6–12 months to attain cognitive benefits. Indeed, although at high risk of bias, the 2 in our review included studies (27,30) that lasted at least 6 months did both find positive effects on physical performance, ADL functioning, and cognition.

Despite the strong promotion of physical exercise for nursing home residents with dementia (4–6), our review revealed heterogeneous results on a wide range of outcomes. The larger studies that contained multimodal exercise interventions seem to suggest a positive effect on physical performance (28,29) and ADL functioning (28,29,36), although not in all studies a significant difference was found. Because of varying outcome measures and a small amount of methodologically sound studies, no effect size could be calculated (Forest plot 2.a). The studies incorporating aerobic interventions (27,30) both found significant positive effects on physical performance, ADL functioning, and cognition. However, because our search strategy identified no more than 2 studies, and both of the studies were at high risk of bias, conclusions about the effectiveness cannot be made.

To some extent, the findings of our review are in line with the existing evidence. A previous systematic review (10) (that included both physical therapists-supervised exercise interventions and non-physical therapist-supervised exercise interventions) found some positive effects, as well as our review. However, that review did not fully report nonsignificant findings, and emphasized positive findings. By emphasizing positive findings, the exercise effects might appear larger than they actually are (17,40).

Regarding the outcome cognition, a prior review (13) identified evidence of a positive effect. Within our review, the 2 studies (28,31) implementing multimodal exercise interventions did not demonstrate a significant effect. However, the 2 studies (27,30) that employed aerobic exercise did show a positive effect. The 2 studies employed aerobic exercise without cognitive tasks, although 1 study (27) did involve social interaction with a caregiver during walking.

In a previous review (14) on exercise interventions for nursing home residents with dementia, significant issues of bias were identified, similar to those found in our own review. Despite that all the RCTs included in our review were published after, the number of RCTs of satisfactory quality remains insufficient to offer a clearer understanding of the subject. Similar to the review of Littbrand et al. (14), we found a lack of transparency about adverse events and the method of assessing them in our included studies. Incomplete or unclear information on the safety of exercise interventions can be harmful. The study by Brett et al. (34) described that recruitment for their study was difficult, because family caregivers were concerned with the safety of the residents, and thought residents were “too old” to exercise. Careful consideration and registration of adverse events can help objectify the risks of an exercise intervention, and inform participants and their caretakers about the (absence of) possible harms.

### Strengths and Limitations

There are some limitations to this systematic review and its evidence base. Due to the risk of bias issues, inconsistency in the findings, and a low number of studies, we could not form a conclusion on the effectiveness of physical therapist-supervised exercise interventions for this particular population. However, this review does describe the current evidence base and its limitations, and thereby forms clear implications for future research. Furthermore, some studies only provided differences in change scores to estimate the effect of the exercise program. This resulted in slight disparities between the study results as reported in the original studies, and the visual representation of the effects in the forest plots based on the postintervention scores. Nevertheless, the forest plots illustrate a valuable aspect of our review, namely the incongruity among the findings of the studies. A strength of this review is that it fulfills all quality criteria of the AMSTAR II (A MeaSurement Tool to Assess systematic Reviews) (41). Our review is also at low risk of bias in the 4 domains assessed by the Risk of Bias in Systematic reviews (42) tool. Not fulfilled criteria are 1.5 (language restrictions) and 4.5 (robustness by funnel plot). We applied language restrictions by only including studies written in English, Spanish, or Dutch. Because almost all studies are published in English, or later translated to English, we think it is unlikely that we missed eligible studies by our language restrictions. We also did not conduct a funnel plot to asses for publication bias. The small amount of included studies (4 studies that used multimodal exercise interventions and 2 studies that used aerobic exercise interventions) give us legitimate reasons to not create a funnel plot (24). We did sufficiently screen our studies on selective reporting, to limit the risk of publication bias.

## Implications

In conclusion, the literature on the characteristics and the effect of physical therapist-supervised exercise interventions in nursing home residents with dementia is heterogeneous and limited. Study length, composition of the intervention, and outcome measures used varied. We included 4 studies that used a multimodal group exercise intervention and 2 studies that used an aerobic exercise intervention, with 3 of the 6 studies at high risk of bias. Exercise effects varied between studies, and were reported on a wide range of health outcomes. No conclusion can be drawn on the effectiveness of exercise interventions based on the studies included in our review. Future studies of high methodological quality can help determine the effects on health outcomes in nursing home residents with dementia.

## Supplementary Material

igae061_suppl_Supplementary_Materials

## Data Availability

The data set and the data script used to create the forest plots are available and can be accessed by contacting the first author. Our review was preregistered in PROSPERO and can be accessed by searching the PROSPERO database for registration number CRD42022351596. All 6 studies preregistered their protocol for ethical purposes at a local or global registration center. The protocols of 3 (28,29,34) of the included studies can be accessed online (see original article for registry information).
