# Supplementary material for: Characteristics and Effectiveness of Physical Therapist-Supervised Exercise Interventions for Nursing Home Residents With Dementia: A Systematic Review
Source: Innov Aging. 2024 Jun 20;8(7):igae061. doi: 10.1093/geroni/igae061 (PMC11287766; doi:10.1093/geroni/igae061)
Supplement: igae061_suppl_Supplementary_Materials [file igae061_suppl_supplementary_materials.docx]

***Innovation in Aging* Supplementary Material: Boer et al.** **Characteristics and effectiveness of physical therapist supervised exercise interventions for nursing home residents with dementia: A systematic review.**

Supplementary Table 1: Complete Search Strategy

| P | ("Nursing Homes"[Mesh] OR "Nursing Homes"[tw] OR "Nursing Home"[tw] OR "Care Homes"[tw] OR "Care Home"[tw] OR "Intermediate Care Facilities"[tw] OR "Intermediate Care Facility"[tw] OR "Skilled Nursing Facilities"[tw] OR "Skilled Nursing Facility"[tw] OR "residential care facilities"[tw] OR "residential care facility"[tw] OR "Residential Facilities"[Mesh] OR "Residential Facilities"[tw] OR "Residential Facility"[tw])  ("Dementia"[Mesh] OR "Dementia"[tw] OR "Dementias"[tw] OR "Dement*"[tw] OR "senile"[tw] OR "amentia"[tw] OR "amentias"[tw] OR "Alzheimer Disease"[tw] OR "Alzheimer"[tw] OR "Alzheimers"[tw] OR "Alzheimer*"[tw] OR "Primary Progressive Aphasia"[tw] OR "Primary Progressive Nonfluent Aphasia"[tw] OR "Creutzfeldt-Jakob Syndrome"[tw] OR "CADASIL"[tw] OR "Diffuse Neurofibrillary Tangles with Calcification"[tw] OR "Frontotemporal Lobar Degeneration"[tw] OR "Pick Disease of the Brain"[tw] OR "Primary Progressive Nonfluent Aphasia"[tw] OR "Huntington Disease"[tw] OR "Huntington"[tw] OR "Huntington*"[tw] OR "Kluver-Bucy Syndrome"[tw] OR "Lewy Body Disease"[tw]) |
| --- | --- |
| I | (("Exercise Therapy"[Mesh] OR "Exercise Therapy"[tw] OR "Exercise Therapies"[tw] OR "Exercise Therap*"[tw] OR "therapeutic exercise"[tw] OR "therapeutic exercises"[tw] OR "Remedial Exercise"[tw] OR "Remedial Exercises"[tw] OR "Rehabilitation Exercise"[tw] OR "Rehabilitation Exercises"[tw] OR "Blood Flow Restriction Therapy"[tw] OR "Continuous Passive Motion Therapy"[tw] OR "Endurance Training"[tw] OR "Muscle Stretching Exercise"[tw] OR "Muscle Stretching Exercises"[tw] OR "Plyometric Exercise"[tw] OR "Plyometric Exercises"[tw] OR "Resistance Training"[tw] OR "Exercise Movement Techniques"[Mesh] OR "Exercise Movement"[tw] OR "Breathing Exercises"[tw] OR "Qigong"[tw] OR "Dance Therapy"[tw] OR "Tai Ji"[tw] OR "Yoga"[tw] OR "Exercise"[Mesh] OR "Exercise"[tw] OR "Exercises"[tw] OR "Circuit-Based Exercise"[tw] OR "Circuit-Based Exercises"[tw] OR "Cool-Down Exercise"[tw] OR "Cool-Down Exercises"[tw] OR "Endurance Training"[tw] OR "Exergaming"[tw] OR "Gymnastics"[tw] OR "High-Intensity Interval Training"[tw] OR "Jogging"[tw] OR "Marathon Running"[tw] OR "Muscle Stretching Exercise"[tw] OR "Muscle Stretching Exercises"[tw] OR "Nordic Walking"[tw] OR "Physical Conditioning"[tw] OR "Plyometric Exercise"[tw] OR "Plyometric Exercises"[tw] OR "Preoperative Exercise"[tw] OR "Preoperative Exercises"[tw] OR "Resistance Training"[tw] OR "Running"[tw] OR "Stair Climbing"[tw] OR "Swimming"[tw] OR "Walking"[tw] OR "Warm-Up Exercise"[tw] OR "Warm-Up Exercises"[tw] OR "psychomotor activation"[tw] OR "psychomotor activat*"[tw] OR "motor activation"[tw] OR "motor activat*"[tw]) |
| M | (randomized controlled trial[pt] OR controlled clinical trial[pt] OR randomized controlled trials[mh] OR random allocation[mh] OR double-blind method[mh] OR single-blind method[mh] OR clinical trial[pt] OR clinical trials[mh] OR "clinical trial"[tw] OR "RCT"[tw] OR "trial"[tw] OR ((singl*[tw] OR doubl*[tw] OR trebl*[tw] OR tripl*[tw]) AND (mask*[tw] OR blind*[tw])) OR "latin square"[tw] OR placebos[mh] OR placebo*[tw] OR random*[tw] OR research design[mh:noexp] OR comparative study[pt] OR evaluation studies[pt] OR follow-up studies[mh] OR prospective studies[mh] OR cross-over studies[mh] OR control[tw] OR controll*[tw] OR prospectiv*[tw] OR volunteer*[tw]) |
| C | - |
| O | - |

Supplementary Table 2: List of excluded studies

|  | Titel | Authors | Year | Reason |
| --- | --- | --- | --- | --- |
| 1 | Effects of a high-intensity exercise program on well-being among older people with dementia living in care facilities: A cluster-randomized trial | Conradsson, M.; Gustafson, Y.; Holmberg, H.; Lindelof, N.; Littbrand, H.; Nordstrom, P.; Rosendahl, E. | 2015 | Abstract |
| 2 | Can the onset of dependency in activities of daily living (ADLs) be delayed in cognitively impaired older adults with short total sleep time? | Lorenz, R. A.; Richards, K. C.; Rose, K. M.; Cole, C. | 2010 | Abstract |
| 3 | Effect of a high-intensity functional exercise program on functional balance: preplanned subgroup analyses of a randomized controlled trial in residential care facilities | Littbrand, H.; Carlsson, M.; Lundin-Olsson, L.; Lindelöf, N.; Håglin, L.; Gustafson, Y.; Rosendahl, E. | 2011 | Cross-sectional analysis of an RCT population |
| 4 | Is the Effect of a High-Intensity Functional Exercise Program on Functional Balance Influenced by Applicability and Motivation among Older People with Dementia in Nursing Homes? | Sondell, A.; Littbrand, H.; Holmberg, H.; Lindelöf, N.; Rosendahl, E. | 2019 | Cross-sectional analysis of an RCT population |
| 5 | Walking Aids Moderate Exercise Effects on Gait Speed in People With Dementia: A Randomized Controlled Trial | Toots, A.; Littbrand, H.; Holmberg, H.; Nordström, P.; Lundin-Olsson, L.; Gustafson, Y.; Rosendahl, E. | 2017 | Cross-sectional analysis of an RCT population |
| 6 | People living in nursing care facilities who are ambulant and fracture their hips: description of usual care and an alternative rehabilitation pathway | Killington, M.; Davies, O.; Crotty, M.; Crane, R.; Pratt, N.; Mills, K.; McInnes, A.; Kurrle, S.; Cameron, I. D. | 2020 | Multi-component intervention |
| 7 | A high-intensity functional weight-bearing exercise program for older people dependent in activities of daily living and living in residential care facilities: evaluation of the applicability with focus on cognitive function | Littbrand, H.; Rosendahl, E.; Lindelöf, N.; Lundin-Olsson, L.; Gustafson, Y.; Nyberg, L. | 2006 | No diagnosis of dementia or no separate analysis |
| 8 | Evaluating the effects of an exercise program (Staying UpRight) for older adults in long-term care on rates of falls: study protocol for a randomised controlled trial | Taylor, L.; Parsons, J.; Taylor, D.; Binns, E.; Lord, S.; Edlin, R.; Rochester, L.; Del Din, S.; Klenk, J.; Buckley, C.; Cavadino, A.; Moyes, S. A.; Kerse, N. | 2020 | No diagnosis of dementia or no separate analysis |
| 9 | Postural stability and quality of life after guided and self-training among older adults residing in an institutional setting | Tuunainen, E.; Rasku, J.; Jäntti, P.; Moisio-Vilenius, P.; Mäkinen, E.; Toppila, E.; Pyykkö, I. | 2013 | No diagnosis of dementia or no separate analysis |
| 10 | A randomized outcome evaluation of group exercise programs in long-term care institutions | Lazowski, D. A.; Ecclestone, N. A.; Myers, A. M.; Paterson, D. H.; Tudor-Locke, C.; Fitzgerald, C.; Jones, G.; Shima, N.; Cunningham, D. A. | 1999 | No diagnosis of dementia or no separate analysis |
| 11 | Why Not a Global Postural Reeducation as an Alternative Therapy Applied to Alzheimer's Patients in Nursing Homes? A Pioneer Randomized Controlled Trial | Todri, J.; Todri, A.; Lena, O. | 2019 | No exercise intervention |
| 12 | The Effects of Exercise on Falls in Older People With Dementia Living in Nursing Homes: A Randomized Controlled Trial | Toots, A.; Wiklund, R.; Littbrand, H.; Nordin, E.; Nordström, P.; Lundin-Olsson, L.; Gustafson, Y.; Rosendahl, E. | 2019 | No outcome data during or after intervention period |
| 13 | A 9-Week Aerobic and Strength Training Program Improves Cognitive and Motor Function in Patients with Dementia: A Randomized, Controlled Trial | Bossers, W. J.; van der Woude, L. H.; Boersma, F.; Hortobágyi, T.; Scherder, E. J.; van Heuvelen, M. J. | 2015 | No PT |
| 14 | A randomised controlled trial testing the impact of exercise on cognitive symptoms and disability of residents with dementia | Stevens, J.; Killeen, M. | 2006 | No PT |
| 15 | Conversation Intervention with Alzheimer's Patients: Increasing the Relevance of Communication | Tappen, R. M.; Williams, C. L.; Barry, C.; Disesa, D. | 2002 | No PT |
| 16 | Effect of Exercise on Behavioral Symptoms and Pain in Patients With Dementia Living in Nursing Homes | Maltais, M.; Rolland, Y.; Vellas, B.; Haÿ, P. E.; Armaingaud, D.; Cestac, P.; Rouch, L.; Cesari, M.; de Souto Barreto, P. | 2019 | No PT |
| 17 | Effect of exercise on mood in nursing home residents with Alzheimer's disease | Williams, C. L.; Tappen, R. M. | 2007 | No PT |
| 18 | Effects of Hand Exercise on Eating Action in Patients With Alzheimer's Disease | Chen, L. L.; Li, H.; Chen, X. H.; Jin, S.; Chen, Q. H.; Chen, M. R.; Li, N. | 2019 | No PT |
| 19 | Effects of Physical Activity in Nursing Home Residents with Dementia: A Randomized Controlled Trial | Henskens, M.; Nauta, I. M.; van Eekeren, M. C. A.; Scherder, E. J. A. | 2018 | No PT |
| 20 | Exercise or Social Intervention for Nursing Home Residents with Dementia: A Pilot Randomized, Controlled Trial | de Souto Barreto, P.; Cesari, M.; Denormandie, P.; Armaingaud, D.; Vellas, B.; Rolland, Y. | 2017 | No PT |
| 21 | Exercise program for nursing home residents with Alzheimer's disease: a 1-year randomized, controlled trial | Rolland, Y.; Pillard, F.; Klapouszczak, A.; Reynish, E.; Thomas, D.; Andrieu, S.; Rivière, D.; Vellas, B. | 2007 | No PT |
| 22 | Physical and functional implications of aquatic exercise for nursing home residents with dementia | Henwood, T.; Neville, C.; Baguley, C.; Clifton, K.; Beattie, E. | 2015 | No PT |
| 23 | REHABILITATION OF MOBILITY AND MOTOR FUNCTION IN NURSING HOME RESIDENTS WITH DEMENTIA | Aizen, E.; Lubosky, E.; Sobeh, S.; Ibrahim, R.; Pressburger, D.; Oliven, R. | 2018 | No PT |
| 24 | The Effect of Exercise and Social Activity Interventions on Nutritional Status in Older Adults with Dementia Living in Nursing Homes: A Randomised Controlled Trial | Maltais, M.; Rolland, Y.; Haÿ, P. E.; Armaingaud, D.; Cestac, P.; Rouch, L.; de Souto Barreto, P. | 2018 | No PT |
| 25 | The effects of movement stimulation on activities of daily living performance and quality of life in nursing home residents with dementia: a randomized controlled trial | Henskens, M.; Nauta, I. M.; Drost, K. T.; Scherder, E. J. | 2018 | No PT |
| 26 | Therapeutic Effects of Exercise Training on Elderly Patients With Dementia: A Randomized Controlled Trial | Liu, I. T.; Lee, W. J.; Lin, S. Y.; Chang, S. T.; Kao, C. L.; Cheng, Y. Y. | 2020 | No PT |
| 27 | Walking and night-time restlessness in mild-to-moderate dementia: a randomized controlled trial | Eggermont, L. H.; Blankevoort, C. G.; Scherder, E. J. | 2010 | No PT |
| 28 | Walking the line: a randomised trial on the effects of a short term walking programme on cognition in dementia | Eggermont, L. H.; Swaab, D. F.; Hol, E. M.; Scherder, E. J. | 2009 | No PT |
| 29 | Improvement of cognitive function after physical movement training in institutionalized very frail older adults with dementia | Thurm, F.; Scharpf, A.; Liebermann, N.; Kolassa, S.; Elbert, T.; Luchtenberg, D.; Woll, A.; Kolassa, I. T. | 2011 | No PT |
| 30 | "Bring me sunshine, bring me (physical) strength": The case of dementia. Designing and implementing a virtual reality system for physical training during the COVID-19 pandemic | Matsangidou, M.; Frangoudes, F.; Hadjiaros, M.; Schiza, E.; Neokleous, K. C.; Papayianni, E.; Avraamides, M.; Pattichis, C. S. | 2022 | No PT |
| 31 | Effects of a 16-week multimodal exercise program on activities of daily living in institutionalized individuals with dementia A multicenter randomized controlled trial | Bezold, J.; Trautwein, S.; Barisch-Fritz, B.; Scharpf, A.; Krell-Roesch, J.; Nigg, C. R.; Woll, A. | 2021 | No PT |
| 32 | Effect of a comprehensive exercise program on function in nursing home residents with Alzheimer's disease | Tappen, R. M.; Roach, K. E.; Touhy, T. A. | 2000 | No PT |
| 33 | Effect of Centella Asiatica and Aerobic Exercise in Older Women With Dementia: A Randomized Controlled Trial | Fitriana, Lisna Anisa; Irma, Darmawati; Nasution, Lina Anisa; Suci Tuty, Putri; Rohaedi, Slamet; Anggadiredja, Kusnandar; Iwan, Setiawan; Nur, Fauziyah; Adnyana, I. Ketut; | 2021 | No PT |
| 34 | Factors predictive of adherence to a non-pharmacological intervention in nursing home research: a substudy of the LEDEN trial | Chrusciel, J.; Letty, A.; Armaingaud, D.; Barreto, P.; Berrut, G.; Rolland, Y.; Sanchez, S. | 2022 | No PT |
| 35 | Effects of 1 Year of Lifestyle Intervention on Institutionalized Older Adults | Magistro, D.; Carlevaro, F.; Magno, F.; Simon, M.; Camp, N.; Kinrade, N.; Zecca, M.; Musella, G. | 2021 | No RCT |
| 36 | Effects of a multicomponent exercise program in institutionalized elders with Alzheimer's disease | Sampaio, A.; Marques, E. A.; Mota, J.; Carvalho, J. | 2019 | No RCT |
| 37 | Effectiveness of individually tailored exercise on functional capacity and mobility in nursing home residents | Bertoncello, C.; Sperotto, M.; Bellio, S.; Pistellato, I.; Fonzo, M.; Bigolaro, C.; Ramon, R.; Imoscopi, A.; Baldo, V. | 2021 | No RCT |

Supplementary Table 3: Outcomes, within and between group differences between baseline and follow-up of the six included studies

|  | **Outcome** | **Within group differences** | **Between group differences^a^** |
| --- | --- | --- | --- |
| **Multimodal exercise interventions** | | | |
| **Physical performance** |  |  |  |
| Brett et al.^34^ | Balance ((M)FR) | median (Q1-Q3): IG 1: pre 12 (8–35) post 28 (17–35) p=0.204; IG 2: pre 16 (9–29) post 26 (12–30) p=0.673; CG: pre 21 (18–31) post 27 (24–33) p=0.271 | p=0.69 |
|  | Balance (FR) | median (Q1-Q3): IG1: pre 12 (0–23) post 18 (11–30) p=0.263; IG2: pre 16 (8–22) post 15 (8–28) p=0.612; CG: pre 15 (12–21) post 13 (7–22) p=0.373 | p=0.60 |
|  | Falls (Number of falls) | median (Q1-Q3): IG 1: pre 0 (0–1) post 0 (0–2) p=0.496; IG2: pre 0 (0–1) post 0 (0–1) p=1.000; CG: pre 0 (0–2) post 1 (0–4) p=0.011 | p=0.02(IG 2 compared to CG) |
|  | Mobility (TUG) | median (Q1-Q3): IG 1 pre 26 (15–36) post 24 (12–29) p=0.045; IG2 pre 26 (17–48) post 27 (19–46) p=0.615; CG pre 23 (17–33) post 21 (16–34) p=0.807 | p=0.53 |
|  | Muscle strength (FTCST) | median (Q1-Q3): IG1 pre 27 (17–41) post 18 (13–44) p=0.130; IG2 pre 25 (20–46) post 21 (15–31) p=0.050; CG pre 22 (18–28) post 26 (18–34) p=0.107 | p=0.63 |
|  | Timed Static Pedalling (TSP) | median (Q1-Q3): IG 1: pre 11 (7–28) post 28 (11–44) p=0.005; IG2: pre 10 (8–29) post 20 (11–34) p=0.040; CG: pre 19 (13–30) post 20 (9–27) p=0.407 | p=0.45 |
|  | Walking speed (Six meter walk test) | median (Q1-Q3): IG1 pre 0.55 (0.46–1) post 0.67 (0.50–1.20) p=0.161; IG2 pre 0.55 (0.38–0.86) post 0.60 (0.33-0.86) p=0.177; CG: pre 0.60 (0.48–0.86) post 0.55 (0.41–0.93) p=0.724 | p=0.53 |
| Telenius et al.^28^ | Balance (BBS) | mean + (SD): IG: pre 34.3 (14.5), post 37.2 (14.0). CG: pre 35.4 (13.7), post 36.6 (14.4) | p=0.02 |
|  | Muscle strength (CST) | mean + (SD): IG: pre 6.0 (3.1), post 7 (3.3). CG: pre 6.2 (2.9), post 6.6 (3.7) | p=0.11 |
|  | Walking speed (Six meter walk test) | mean + (SD): IG: pre 0.5 (0.2), post 0.5 (0.2). CG: pre 0.5 (0.2), post 0.5 (0.3) | p=0.86 |
| Toots et al.^29^ | Balance (BBS) | MD + (SE): IG: 2.39 (0.88) CG: -1.82 (0.86) | MD + (95% CI): 4.20 (1.79-6.61) p=<0.001 |
| Toots et al.^32^ | Walking speed (Backward walking speed) | MD + (SE): with or without WA: IG: 0.005 (0.013); CG: 0.000 (0.013); without WA: IG: 0.026 (0.018); CG: -0.004 (0.017) | MD + (95% CI): with or without WA: 0.005 (-0.031-0.041) p=0.788; without WA: 0.030 (–0.019, 0.079) p=0.231 |
| **ADL functioning** |  |  |  |
| Littbrand et al.^36^ | ADL performance (BI) | MD + (SD): IG: -0.13 (2.11) p=0.67; CG: -1.10 (2.28) p=0.001 | MD (95% CI): 1.13 (0.12-2.13) p=0.03 |
| Telenius et al.^28^ | ADL performance (BI) | mean + (SD): IG: pre 13.6 (3.5), post 13.7 (3.6); CG: pre 13.4 (3.6), post 12.7 (4.1) | p=0.085 |
| Toots et al.^29^ | ADL performance (BI) | MD + (SE): IG: -0.79 (0.31); CG: -1.39 (0.30) | MD + (95% CI): 0.60 (-0.24-1.44) p=0.16 |
|  | ADL performance (FIM) | MD + (SE): IG: -3.10 (1.07); CG: -4.44 (1.04) | MD + (95% CI): 1.34 (-1.56-4.25) p=0.36 |
| **Cognition** |  |  |  |
| Telenius et al.^28^ | Global cognition (MMSE) | mean + (SD): IG: pre 15.6 (5.0), post 15.5 (5.5); CG: pre 15.8 (5.0), post 15.2 (5.4) | p=0.69 |
| Toots et al.^31^ | Global cognition (MMSE) | MD + (SE): IG: –1.15 (0.41); CG: –0.93 (0.4) | MD + (95% CI): -0.27 (-1.4-0.87) p=0.644 |
|  | Executive functioning (VF) | MD + (SE): IG: –0.74 (0.32); CG: –0.21 (0.32) | MD + (95% CI): -0.53 (-1.42-0.35) p=0.241 |
|  | Global cognition (ADAS-Cog) | MD + (SE): IG: 1.51 (1.06); CG: 2.55 (1.07) | MD + (95% CI): -1.04 (-4-1.92) p=0.491 |
| **Psychological wellbeing** |  |  |  |
| Bostrom et al.^33^ | Depression (GDS-15) | IG: MD + (95% CI) 0.03 (–0.53, 0.59) p=0.91; CG MD + (95% CI) 0.08 (–0.49, 0.64) p=0.78 | MD + (95% CI) -0.05 (-0.84, 0.75) p=0.91 |
|  | Depression (MADRS) | IG: MD + (95% CI) 0.40 (–0.77, 1.57) p=0.50; CG: MD + (95% CI) 0.33 (–0.85, 1.52) p=0.58 | MD + (95% CI) 0.06 (-1.60, 1.73) p=0.94 |
| Brett et al.^35^ | Agitation (CMAI) | IG 1: pre 28 (9) post 26 (10) p=0.03; IG 2: pre 26 (9) post 24 (8) p=0.23; CG: pre 36 (8) post 32 (8) p=0.02 | p>0.05 |
| Conradsson et al.^37^ | Depression (GDS-15) | MD + (SD): IG: 0.03 (2.3) p=0.92; CG: -0.10 (1.9) p=0.62 | MD + (95% CI): -0.33 (-1.20, 0.55) p=0.46 |
|  | Morale (PGCMS) | MD + (SD): 0.35(2.7) p=0.25 CG: 0.02 (2.3) p=0.93 | MD + (95% CI): 1.12 (0.09 to 2.16) p=0.03 |
| Telenius et al.^28^ | Agitation (NPI-Agitation) | mean + (SD): IG: pre 1.7 (2.1), post 1.5 (2.2); CG: pre 1.3 (1.7), post 1.7 (2.3) | p=0.07 |
|  | Apathy (NPI-Apathy) | mean + (SD): IG: pre 0.5 (0.8), post 0.3 (0.6); CG: pre 0.39 (0.7), post 0.4 (0.8) | p=0.048 |
|  | Neuropsychiatric symptoms (NPI) | mean + (SD): IG: pre 5.8 (5.9), post 5.1 (6.0); CG: pre 4.8 (4.6) , post 5.4 (6.5) | p=0.17 |
|  | Affect (NPI-depression and anxiety) | mean + (SD): IG: pre 1.1 (1.4), post 1.0 (1.4); CG: pre 0.8 (1.3), post 1.0 (1.4) | p=0.31 |
|  | Depression (Cornell scale) | mean + (SD): IG: pre 4.7 (4.6), post 3.8 (5.2); CG: pre 4.9 (4.3), post 3.8 (3.8) | p= 0.39 |
|  | Quality of life (Qualid) | mean + (SD): IG: pre 18.3 (6.1), post 17.1 (7.0); CG: pre 17.7 (5.5), post 17.4 (6.6) | p= 0.97 |
| **Aerobic exercise interventions** | | | |
| **Physical performance** |  |  |  |
| Cancela et al.^30^ | Mobility (TUG) | Mean difference + 95%CI: IG: -2.11 (-3.86,-0.36); CG -0.56 (-1.45, 0.33) | p=0.03 |
| Venturelli et al.^27^ | Walking distance (6mwt) | mean + (SD): IG: pre 245 (31) post 294 (49) p<0.05 CG: pre 238 (47) post 168 (34) p<0.05 | p<0.05 |
| **Psychological wellbeing** |  |  |  |
| Cancela et al.^30^ | Depression (Cornell Scale) | Mean difference + 95%CI: IG: 1.84 (-0.80, 4.48); CG: -2.71 (-3.52, -1.91) | p=0.22 |
|  | Neuropsychiatric symptoms (NPI) | Mean difference + 95%CI: IG: -0.84 (-3.76, 2.08); CG: 4.60 (1.96, 7.24) | p=0.08 |
| **ADL functioning** |  |  |  |
| Cancela et al.^30^ | ADL functioning (Katz Index) | Mean difference + 95%CI: IG: 0.22 (-0.05, 0.49); CG: -0.25 (-0.36, -0.14) | p=0.03 |
| Venturelli et al. (2011) | ADL functioning (BI) | mean + (SD): IG: pre 34 (4) post 42 (4) p<0.05; CG: pre 35 (6) post 32 (6) p>0.05 | p=0.003 |
| **Cognition** |  |  |  |
| Cancela et al.^30^ | Global cognition (MEC -Spanish MMSE) | Mean difference + CI: IG: 0.09 (-0.32, 0.60); CG: -2.11 (-2.90,-1.32) | p=0.01 |
|  | Memory (FOME) | Mean difference + 95%CI: IG: 1.99 (-1.93, 4.33); CG: -1.31 (-3.57, 7.95) | p=0.01 |
| Venturelli et al.^27^ | Global cognition (MMSE) | mean + (SD): IG pre 13 (2) post 12 (2) p=>0.05; CG: pre 12 (2) post 6 (2) p<0.05 | p<0.001 |
| **Others** |  |  |  |
| Venturelli et al.^27^ | (Fasting) Glycemia (mg/dl-1) | mean + (SD): IG: pre 94(5) post 90(3) p=0.784; CG: pre 92(5) post 93(5) p=0.345 | p=0.143 |
|  | Systolic blood pressure (mmHg) | mean + (SD): IG: pre 132(10) post 126(8) p=0.05; CG: pre 133(6) post 135(7) p=0.368 | p=0.108 |
|  | Diastolic blood pressure (mmHg) | mean + (SD): IG: pre 84(5) post 82(3) p=0.550; CG: pre 84(3) post 84(3) p=0.334 | p=0.376 |
| ^a^Mean difference with 95% CI or p-value of test of between group difference if mean difference with 95% CI was not available.  (M)FR=(Modified) Functional Reach test, TUG= Timed Up and Go test, FTSTS= Five-Times-Sit-to-Stand, TSP= timed static pedalling, BBS= Berg Balance Scale, CST= Chair Stand Test (30 sec), MD= Mean difference, WA= walking aid, GDS 15= Geriatric Depression Scale 15-item version, MADRS= Montgomery–Åsberg Depression Rating Scale, CMAI= Cohen-Mansfield Agitation Inventory, PGCMS= Philadelphia Geriatric Center Morale Scale, BI=Barthel Index, FIM= Functional Independence Measure, MMSE= Mini-Mental State Examination, NPI-Q= The Neuropsychiatric Inventory questionnaire, QUALID= Quality of life in late-stage dementia scale, VF= Verbal fluency, ADAS-COG= Alzheimer’s Disease Assessment Scale-Cognitive Subscale, 6mwt= six minute walking test, FOME=Fuld Object Memory Evaluation, mg/dl=milligrams per decilitre, mmHg= millimetre of mercury | | | |

Supplementary Table 4: Certainty in the evidence according to the GRADE approach

| **Multimodal exercise interventions** | **No of studies** | **N total** | **Study design and execution** | **Inconsistency** | **Indirectness** | **Imprecision** | **Publication bias** | **GRADE** |
| --- | --- | --- | --- | --- | --- | --- | --- | --- |
| **Physical performance** |  |  |  |  |  |  |  |  |
| Balance | 3 | 355 | -2 (one study at high risk of bias) | -1 (2 in favor of intervention, 1 no effect) | -1 (differences in intervention duration and frequency, differences in control groups) | -1 (one study with a large confidence interval) | - | Very low |
| Walking speed | 3 | 387 | -2 (one study at high risk of bias) | - | -2 (differences in intervention duration and frequency, differences in control groups. difference in outcome measure) | -1 (one study with a large confidence interval) | - | Very low |
| **ADL functioning** |  |  |  |  |  |  |  |  |
| ADL functioning | 3 | 421 | -1 (one study with RoB concerns) | -2 (1 in favor of intervention, 2 no effect) | - | - | - | Very low |
| **Psychological wellbeing** |  |  |  |  |  |  |  |  |
| Depression | 3 | 314 | -1 (one study with RoB concerns) | - 1 (different directions of effect) | - | -1 (one study with a large confidence interval) | - | Very low |
